# A Longitudinal Twin Study of Skewed X Chromosome-Inactivation

**DOI:** 10.1371/journal.pone.0017873

**Published:** 2011-03-22

**Authors:** Chloe Chung Yi Wong, Avshalom Caspi, Benjamin Williams, Renate Houts, Ian W. Craig, Jonathan Mill

**Affiliations:** 1 MRC Social, Genetic and Developmental Psychiatry Centre, Institute of Psychiatry, King's College London, London, United Kingdom; 2 Departments of Psychology and Neuroscience, Psychiatry and Behavioral Sciences, Institute for Genome Sciences and Policy, Duke University, Durham, North Carolina, United States of America; Victor Chang Cardiac Research Institute, Australia

## Abstract

X-chromosome inactivation (XCI) is a pivotal epigenetic mechanism involved in the dosage compensation of X-linked genes between males and females. In any given cell, the process of XCI in early female development is thought to be random across alleles and clonally maintained once established. Recent studies, however, suggest that XCI might not always be random and that skewed inactivation may become more prevalent with age. The factors influencing such XCI skewing and its changes over time are largely unknown. To elucidate the influence of stochastic, heritable and environmental factors in longitudinal changes in XCI, we examined X inactivation profiles in a sample of monozygotic (MZ) (n = 23) and dizygotic (DZ) (n = 22) female twin-pairs at ages 5 and 10 years. Compared to MZ twins who were highly concordant for allelic XCI ratios, DZ twins showed much lower levels of concordance. Whilst XCI patterns were moderately stable between ages 5 and 10 years, there was some drift over time with an increased prevalence of more extreme XCI skewing at age 10. To our knowledge, this study represents the earliest longitudinal assessment of skewed XCI patterns, and suggests that skewed XCI may already be established in early childhood. Our data also suggest a link between MZ twinning and the establishment of allelic XCI ratios, and demonstrate that acquired skewing in XCI after establishment is primarily mediated by stochastic mechanisms. These data have implications for our understanding about sex differences in complex disease, and the potential causes of phenotypic discordance between MZ female twins.

## Introduction

Unlike males who have only one X chromosome, females carry both a maternally- and paternally-inherited X chromosome. To ensure dosage compensation of X-linked genes with males, many loci on the X chromosome are allelically inactivated in females. This X chromosome inactivation (XCI) is associated with hypermethylation of CpG islands, a process regulated by the X-chromosome-inactivation centre [1]. XCI occurs early in female embryonic development, and is believed to be stochastically-determined and clonally maintained once established [1]. As a result of XCI, heterozygous females are hemizygous mosaics for the expression of most X-linked genes, with one population of cells expressing maternally inherited alleles and the other population expressing paternally inherited alleles [2]. Recent studies, however, suggest that XCI might not be truly equal across both alleles, with a bias towards silencing of either the paternally- or maternally-inherited allele leading to allelically skewed XCI patterns [3]. Skewing in favour of either the maternally- or paternally-inherited X chromosomes is relatively common in adult females, with about 35% of individuals having greater than 20% skewed XCI in either direction [3]. Extreme skewing (>40%) has been observed in about 7% of females and becomes more prevalent with increasing age [4], [5], [6], [7], [8], [9], [10], [11]. To date, most studies of age-associated changes in XCI have been based on cross-sectional comparisons of female samples of different ages. Only a number of limited intra-individual studies have been performed; these indicate that while XCI patterns appear somewhat stable over time, there is increased prevalence of significantly-skewed XCI in older females [12], [13]. No study has yet assessed longitudinal changes in XCI in females during childhood, and the specific factors (stochastic, heritable, environmental) controlling allelic biases in XCI, as well as changes in such biases over time, are largely unknown. In the present study, we assessed allelic patterns of XCI in both monozygotic (MZ) and dizygotic (DZ) female twin-pairs using two successive DNA samples taken 5 years apart during childhood development from the same set of individuals. Our goal was to ascertain the influence of stochastic, heritable and environmental factors on skewed X-inactivation, and document changes in XCI patterns over time.

## Results

### Distribution of X-Inactivation Patterns in female children at ages 5 & 10 years

**Figure 1:**
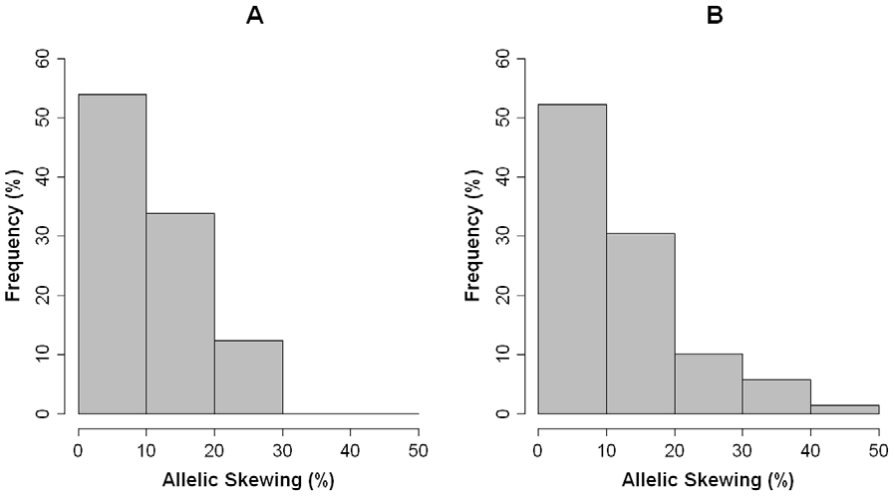
Allelic skewing of female children at ages 5 and 10 years as ascertained by the HUMARA assay performed in triplicate. Allelic skewing of female children at (***A***) age 5 and (***B***) age 10. Allelic skewing is defined as the percentage deviation from the presumed ‘normal’ 50∶50 XCI patterns and is assigned to 5 “bins” representing increments of 10%. More instances of extreme allelic XCI skewing are observed at age 10 than age 5. *XCI = X-Chromosome Inactivation*.

We observed similar allelic patterns of XCI between MZ and DZ twins at ages 5 and 10 (see **Table 1**) ; the means of the distributions for ages 5 and 10 years were 48∶52 (allele 1: allele 2), i.e. 2% skewing, and 47∶53 (3% skewing), respectively. However there was a wider range of XCI ratio at age 10 (9∶91 to 84∶16) compared to age 5 (22∶78 to 77∶23), and an increased prevalence of females with allelic skewing greater than 20% (i.e. XCI ratio >70∶30 or <30∶70) at age 10 (17.4%) compared to age 5 (12.3%). Whilst no individual demonstrated allelic skewing greater than 30% at age 5, 7.3% of individuals at age 10 had skewed XCI ratios greater than 30%, with 1.5% demonstrating allelic skewing greater than 40% (**Figure 1**).

**Table 1:** Skewed X-inactivation ratios (deviation from 50∶50) in females at ages 5 and 10 years (The percentage reported is cumulative for each group).

			Percentage of Sample with Allelic Skewing of		
Age	Zygosity	Mean (SD)	>20%	>30%	>40%
**5**	**ALL**	**48∶52** (13.4)	**12.3 (n = 8)**	**0**	**0**
	**MZ**	**47∶53** (13.8)	**12.9 (n = 4)**	**0**	**0**
	**DZ**	**49∶51** (13.2)	**11.8 (n = 4)**	**0**	**0**
**10**	**ALL**	**47∶53** (14.8)	**17.4 (n = 12)**	**7.3 (n = 5)**	**1.5 (n = 1)**
	**MZ**	**47∶53** (16.7)	**23.5 (n = 8)**	**8.8 (n = 3)**	**2.9 (n = 1)**
	**DZ**	**47∶53** (12.9)	**11.4 (n = 4)**	**5.7 (n = 2)**	**0**

### Stability of X-inactivation

**Figure 2:**
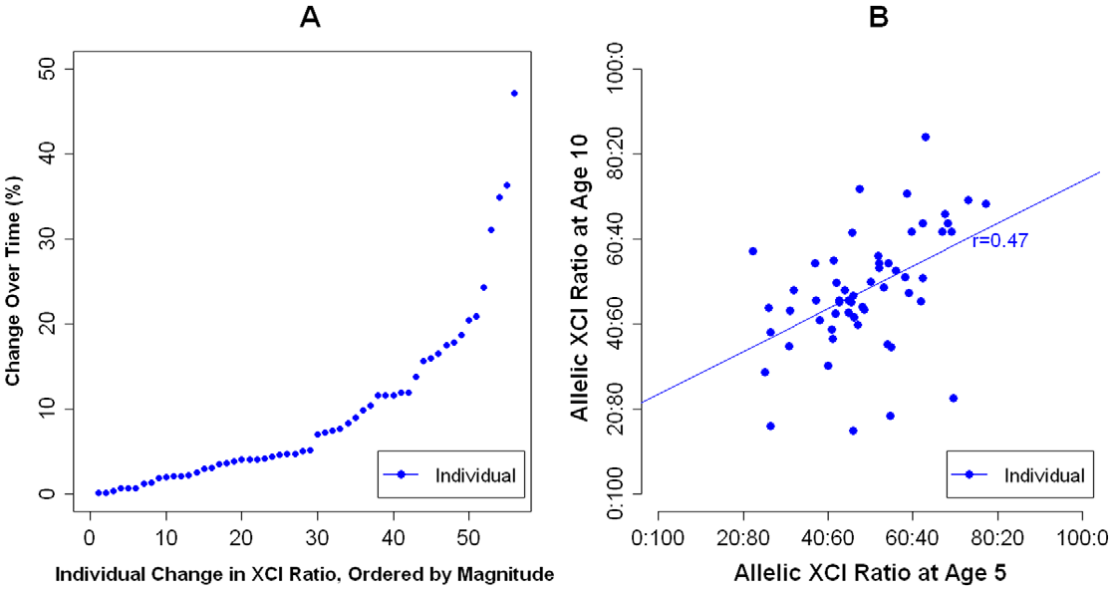
Longitudinal analysis of allelic XCI ratios in female children over early childhood development. (***A***) Individual changes in absolute XCI ratio between ages 5 and 10 years. (***B***) Inter-individual stability correlation for XCI ratio, between ages 5 and 10 years. *XCI = X-Chromosome Inactivation*.

A wide range of change in allelic XCI patterns between ages 5 and 10 years was observed (**Figure 2A**). While the majority (74.9%) of the sample demonstrated highly stable patterns of XCI between ages 5 and 10 years, with a shift of less than 10% in XCI ratio, 11.9% showed a skew of greater than 20% with 6.8% of the samples changing by greater than 30% during this period. A significant inter-individual stability in XCI ratio over time was observed (r = 0.47; p = 0.01) (**Figure 2B**). Furthermore, the allelic direction of XCI skewing was stable across ages 5 and 10; 92.3% of individuals with allelic XCI skewing of greater than 10% (i.e. XCI ratio >60∶40 or <40∶60) showed the same direction of allelic skewing across both ages.

### MZ and DZ within-twin correlations for skewed XCI

**Figure 3:**
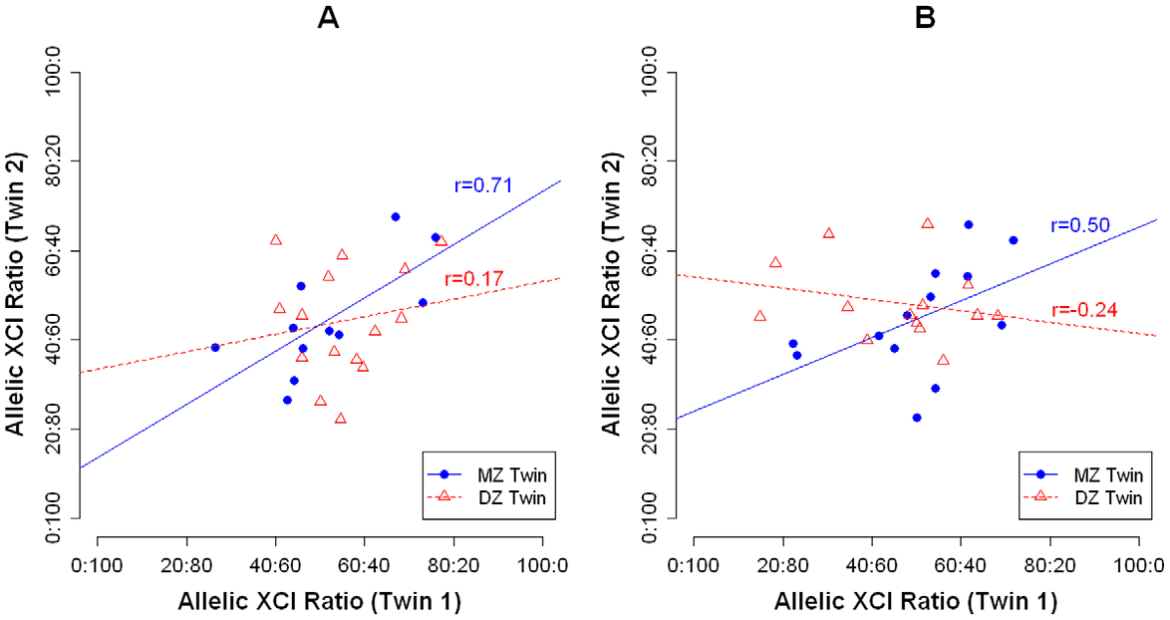
Allelic XCI ratios of MZ and DZ twin pairs at ages 5 and 10 years. (***A***) MZ and DZ twin correlations for XCI ratio at age 5. (***B***) MZ and DZ twin correlations for XCI ratio at age 10. *XCI = X-Chromosome Inactivation*; *MZ = Monozygotic*; *DZ = Dizygotic*.

**Figures 3A and 3B** show the XCI ratio of each twin versus her co-twin at ages 5 and 10 years, respectively. At both age 5 and age 10, twin concordances for allelic XCI ratio were substantial for MZ-twins (r = 0.71, p = 0.01 and r = 0.50, p = 0.08 for ages 5 and 10 years, respectively) and negligible for DZ-twins (r = 0.17, p = 0.56 and r = −0.24, p = 0.41 for ages 5 and 10 years, respectively). We observed little concordance in within-pair intra-individual change correlations for XCI ratio in both MZ (r = 0.20; p = 0.64) and DZ twin-pairs (r = −0.45; p = 0.14), suggesting that alterations in XCI patterns between ages 5 and 10 years are not influenced by factors common to both members of a twin-pair (i.e. heritable or shared environmental influences).

## Discussion

XCI is a fundamental mammalian dosage compensation process, regulated by epigenetic mechanisms, that operates to ensure the equal expression of X-linked genes between males and females. In this study, we employed a genetically informative approach using female twin-pairs to assess allelic XCI patterns across two ages (age 5 and age 10) during childhood development. The mean allelic XCI ratio across all individuals resided near 50∶50 at both ages, as expected given the presumed stochastic nature of XCI. Interestingly, however, we find that allelic XCI skewing of greater than 20% (i.e. XCI ratio >70∶30 or <30∶70) is already apparent in some children by age 5 (12.3% of all female children) and becomes more prevalent by age 10 (17.4% of all female children). These data support the conclusions from previous analyses demonstrating an increase in XCI skewing with age [4], [5], [6], [7], [8], [9], [10], [11], [12], [13], [14]. This study, to our knowledge, represents the earliest longitudinal assessment of skewed XCI patterns, and suggests that skewed XCI may already be established in early childhood; previous data show that the prevalence of marked (>20%) XCI skewing increases to greater than 35% during adulthood [3]. Whilst the degree of skewing was subject to some change over time, the direction of XCI skewing remained largely stable between ages 5 and 10. 92.3% of individuals with allelic XCI skewing of greater than 10% (i.e. XCI ratio >60∶40 or <40∶60) showed the same direction of allelic skewing across both ages.

Our use of a genetically informative twin design allowed us to elucidate the factors (stochastic, heritable, or environmental) contributing to allelic skewing of XCI. Twin correlations for XCI patterns were high for MZ twins (r = 0.71 at age 5 and r = 0.50 at age 10, respectively) but very low for DZ twins at both ages (r = 0.17 at age 5 and r = −0.24 at age 10, respectively), concurring with existing data [11], [14]. Quantitative genetic analyses traditionally interpret such a dramatically higher concordance rate in MZ twins compared to DZ twins as indicating the contribution of a large non-additive genetic effect to XCI patterns [11]. An alternative explanation is mechanistic. The initiation of XCI is believed to be a largely stochastic biological process occurring at the time of, or just before, the process of MZ twinning [15], potentially explaining the higher similarity in XCI profiles within MZ twin-pairs. The relationship between X-inactivation and the timing of MZ-twinning is supported by previous research demonstrating that X-inactivation patterns are virtually identical in monoamniotic-monochorionic-MZ twin pairs, highly similar in diamniotic-monochorionic-MZ twin pairs and notably different in dichorionic-MZ twin pairs [16], [17].

Because DZ twins originate from individually fertilized embryos that are subjected to independently established XCI patterns, it is not surprising that our data concur with other studies highlighting minimal correlation between DZ twin-pairs. Once XCI patterns are established they are subject to stochastic drift over embryonic (and later, post-natal) development [5]. Such random changes in XCI explain the observed reduction in MZ correlation observed between ages 5 and 10 years in our data (**Figure 3**), and the increased level of MZ twin discordance seen with advancing age in other published data [11]. Our observation of little MZ and DZ twin concordances for *change* in XCI patterns between ages 5 and 10 years further supports the notion that alterations in XCI ratios in early development are primarily regulated by stochastic mechanisms [18], [19].

Despite the overall high MZ twin correlations in allelic XCI ratios observed at both time points, there is still noticeable XCI discordance among genetically identical individuals: the average within-twin-pair difference in allelic XCI ratio was 10.8% (range from 0.71% to 24.7%) at age 5 and 10.7% (range from 0.49% to 27.7%) at age 10. The observed discordance in XCI patterns within MZ twin-pairs adds to the growing body of evidence of how genetically identical individuals can be epigenetically different [20], [21], [22], [23]. Such MZ twin differences in allelic XCI ratios may have relevance for etiological studies of disease. Marked discordance for skewed XCI has been observed in female MZ twin pairs discordant for X-linked gene disorders including Duchenne muscular dystrophy [24], fragile X mental retardation [25] and even complex non-Mendelian disorders such as bipolar disorder [26], [27].

It is important to note that our study has several limitations and that the findings reported here require replication in additional longitudinal sample cohorts. First, our study assessed longitudinal changes in XCI using DNA obtained from buccal cells, which may not necessarily be reflective of patterns observed in other cell types. However, whilst it is likely that there are tissue-specific factors influencing XCI, data from our group and others indicate that, as expected for a key epigenetic pattern established early in development, allelic patterns of XCI are strongly conserved across tissues obtained from the same individual, including comparisons of blood and saliva [28], blood and buccal cells [16], [26], and blood and brain [29]. Second, our sample size is relatively small for a twin study and this has precluded the use of quantitative twin model fitting. Future studies will extend these analyses to a larger number of twin-pairs. Third, we do not have access to our twins' parental DNA, precluding us from calculating the direction (towards the paternal or maternal X chromosome) of XCI skewing. Finally, the amniotic and chorionic data of our twins are unavailable, which prevent us from testing hypotheses about how amnionicity and chorionicity influence XCI concordance [16], [17]. Future studies investigating longitudinal skewed X-inactivation patterns in MZ twins of known amnionicity and chorionicity (indicating at what developmental time the twinning event happened) across additional ages will allow us to gain further insight into the relationship between MZ twinning and XCI, and elucidate the factors that underlie acquired skewing.

To conclude, this study assessed longitudinal changes in skewed XCI patterns in both MZ- and DZ-twin pairs during the course of early childhood development. Findings from our genetically informative study support the notion of a link between MZ twinning and the establishment of allelic XCI ratios, and suggest that acquired skewing in XCI after establishment is primarily mediated by stochastic mechanisms. A better understanding of the factors underlying skewed XCI will provide insight into the role it plays in mediating sex differences in complex disease and phenotypic discordance between MZ female twins.

## Methods

### Subjects

Samples were obtained from Caucasian MZ and DZ twin-pairs enrolled in the Environmental Risk (E-Risk) Longitudinal Twin Study, which has been described in detail elsewhere [30]. In brief, E-Risk investigates how genetic and environmental factors shape children's development. For our XCI analyses, we selected female children, comprising 23 MZ twin-pairs and 22 DZ twin pairs for whom DNA was available at both ages 5 and 10 years. DNA samples (total n = 180) were collected from buccal cells using an established method yielding high molecular weight genomic DNA [31]. All DNA samples were tested for degradation and purity; any degraded or impure samples, as well as those uninformative (i.e. homozygous) for the XCI assay were excluded from analysis (25 and 21 DNA samples were excluded at ages 5 and 10 years, respectively). Ethical approval was granted by the Joint South London and Maudsley and the Institute of Psychiatry Research Ethics Committee and written informed consent were given by all subjects.

### Determination of X-inactivation pattern

Allelic XCI ratios were determined by examining DNA methylation in the vicinity of a polymorphic (CAG)_n_ repeat in the first exon of the human androgen receptor (*AR*) gene as described previously [26], [32]. DNA methylation across this region of the *AR* gene is highly correlated with X-inactivation status [32] and the human androgen receptor assay (HUMARA) is a widely used clinical assay for determining XCI status. In brief, 150 ng of genomic DNA was divided into three aliquots and incubated with *Hpa*II, *Msp*I or water. Digested DNA was amplified using primers (one fluorescently labeled) flanking the polymorphic (CAG)_n_ repeat in the first exon of the *AR* gene (F: 5′-FAM-GCTGTGAAGGTTGCTGTTCCTCAT-3′ and R:5′-TCCAGAATCTGTTCCAGAGCGTGC-3′). Fluorescently labeled products were separated on an automated DNA sequencer (Applied Bioscience, Foster City, CA, USA) to accurately quantify the peak heights for each allele. Allelic XCI ratios were calculated as the normalized ratio of peak heights of the shorter to longer alleles of the *Hpa*II digest to those observed in a water (mock) digest.

The formulae used to calculate XCI ratio were as follows (see also [16]):

Ratio in mock digestion (R_m_) = allele 1 peak height/allele 2 peak height
Ratio in *Hpa*II digestion (R_h_) = allele 1 peak height/allele 2 peak height
Normalized ratio (R_n_) = R_h_/R_m_
Percentage of inactivation of allele 1 = [R_n_/(R_n_+1)]×100.

Digestion efficiency was confirmed using the *Msp*I digest (which cleaves irrespective of DNA methylation status) on the same DNA samples. All samples were randomized in the experiment and each sample was independently processed in triplicate for the entire experiment to control for potential technical bias. The average XCI scores across all readings were calculated for each individual and reported in this study.
